# Mitochondria-Related Transcriptome Characterization Associated with the Immune Microenvironment, Therapeutic Response and Survival Prediction in Pancreatic Cancer

**DOI:** 10.3390/ijms24043270

**Published:** 2023-02-07

**Authors:** Jia Dong, Jiang Liu, Bo Zhang, Chen Liang, Jie Hua, Qingcai Meng, Miaoyan Wei, Wei Wang, Xianjun Yu, Jin Xu

**Affiliations:** 1 Department of Pancreatic Surgery, Shanghai Cancer Centre, Fudan University, Shanghai 200032, China; 2 Department of Oncology, Shanghai Medical College, Fudan University, Shanghai 200032, China; 3 Shanghai Pancreatic Cancer Institute, Shanghai 200032, China; 4 Pancreatic Cancer Institute, Fudan University, Shanghai 200032, China

**Keywords:** pancreatic cancer, mitochondria-related genes, survival, chemotherapeutic response

## Abstract

(1) Background: Pancreatic cancer (PC) is one of the most lethal tumors. Mitochondrial dysfunction has been reported to be involved in cancer development; however, its role in PC has remained unclear. (2) Methods: The differentially expressed NMGs were selected between PC and normal pancreatic tissue. The NMG-related prognostic signature was established by LASSO regression. A nomogram was developed based on the 12-gene signature combined with other significant pathological features. An extensive analysis of the 12 critical NMGs was performed in multiple dimensions. The expression of some key genes was verified in our external cohort. (3) Results: Mitochondria-related transcriptome features was obviously altered in PC compared with normal pancreas tissue. The 12-NMG signature showed good performance in predicting prognosis in various cohorts. The high- and low-risk groups exhibited notable diversity in gene mutation characteristics, biological characteristics, chemotherapy response, and the tumor immune microenvironment. Critical gene expression was demonstrated in our cohort at the mRNA and protein levels and in organelle localization. (4) Conclusions: Our study analyzed the mitochondrial molecular characterization of PC, proving the crucial role of NMGs in PC development. The established NMG signature helps classify patient subtypes in terms of prognosis prediction, treatment response, immunological features, and biological function, providing a potential therapeutic strategy targeting mitochondrial transcriptome characterization.

## 1. Introduction

According to the World Health Organization’s estimates for 2022, pancreatic cancer (PC) ranks as the world’s third most lethal malignancy. PC shows an increasing incidence and a poor five-year survival rate of less than 10%, which is a challenging situation [1]. Typically, the vast majority of patients are diagnosed with PC at advanced stages, usually accompanied by distant metastasis, and fewer than 20% of patients have the chance to receive radical surgery [2]. The early asymptomatic features resulting from the deep anatomical location and limited treatment methods with unclear efficiency make PC a severe public health challenge [3]. With the emergence of new tumor treatment methods, including antiangiogenic drugs and immunotherapy, it is crucial to discover new biomarkers to identify patients who may be able to benefit from specific therapeutic strategies [4].

Mitochondria are acclaimed as the “powerhouse” of eukaryotic cells, influencing the cell fate via energy production and material metabolism [5]. Many complex energy metabolic reactions occur in mitochondria, including the tricarboxylic acid cycle (TCA cycle), oxidative phosphorylation (OXPHOS), and electron transport chain (ETC), maintaining the expected growth of cells [6]. Research has proven that mitochondrial dysfunction can cause adverse effects on reactive oxygen species (ROS) homeostasis, calcium homeostasis, endoplasmic reticulum (ER) stress, inflammation, and cell apoptosis [7]. On the one hand, the elevated ROS resulting from mitochondrial dysfunction could cause DNA damage and genetic instability [8,9]; on the other hand, excessive ROS inactivates key enzymes such as NADH dehydrogenase, leading to mitochondrial DNA (mtDNA) destruction and mutations [10]. There are many pathologies associated with mitochondrial metabolic reprogramming, including tumors, diabetes, cardiomyopathy, and neurodegenerative diseases [11].

An increasing number of studies have explored the critical role of mitochondria in malignancies, including PC. To satisfy the excessive energy demand, pancreatic cancer cells change mitochondria-related gene expression profiles and reshape the metabolic energy pattern [12]. Through “metabolic reprogramming”, cancer cells seize sufficient energy to maintain aggressive behaviors, including angiogenesis and metastasis. Relevant biochemical processes also promote pancreatic cancer. OXPHOS enhances the chemoresistance, invasiveness, and immune-evasive properties of PC stem cells [13]. The tricarboxylic acid (TCA) cycle plays a central role in the oxidative phosphorylation process, and an abnormal TCA cycle alters the redox balance and metabolite production in the process of cancer development [14,15]. The electron transport chain (ETC) can fuel the oxidative phosphorylation process by increasing electron flow or affecting the structure and composition of the ETC [16], producing more reactive oxygen species (ROS) to promote oncogenic growth and exert its oncogenic effects [17].

Except for the 37 genes encoded by the mitochondrial genome (mtDNA), most mitochondrial proteins are encoded by the nuclear genome, synthesized in the cytosol, and then transported to the mitochondria [18]. Altered mitochondrial gene profiles lead to changes in the number of specific proteins and promote tumor progression. Mitochondrial proteins play significant roles in the reprogramming of PC. Mitochondrial uncoupling protein 2 (UCP2) could help to transport aspartate into the cytosol to reshape KRAS^mut^ glutamine metabolism [19]. YME1L is a type of i-AAA protease in the inner mitochondrial membrane that mediates mitochondrial proteolytic rewiring to support cell growth in hypoxia [20]. The mitochondrial protein UQCRC1 has been proven to promote PC development by increasing oxidative phosphorylation (OXPHOS) and ATP production [21]. The mitochondrial fission GTPase dynamin-related protein 1 (Drp1) influences glycolytic and oxidative metabolism to promote the development of the KRAS-driven PC model [22]. Mitochondrial calcium uniporter (MCU) supports the invasive phenotype and metabolic stress resistance of PC cells by activating the Keap1–Nrf2 antioxidant signaling pathway [23]. However, most studies have focused on a particular gene rather than the integrated cluster of mitochondria-related genes. The function of mitochondria-related genes in pancreatic cancer is worthy of further exploration.

Therefore, we carried out a systematic exploration of the mitochondrial transcriptome land scape in pancreatic cancer and established the NMG signature to conduct risk stratification of patients. Moreover, the differential gene expression, mutation landscape, tumor microenvironment, and chemotherapy response were analyzed in the two risk groups. The prognostic value of NMGs was confirmed in the training set and external verification sets. We hope our research will be helpful for clinical decision making and precise therapy of pancreatic cancers.

## 2. Results

### 2.1. Mitochondrial Disorder Plays a Crucial Role in PC Development

**Figure 1:**
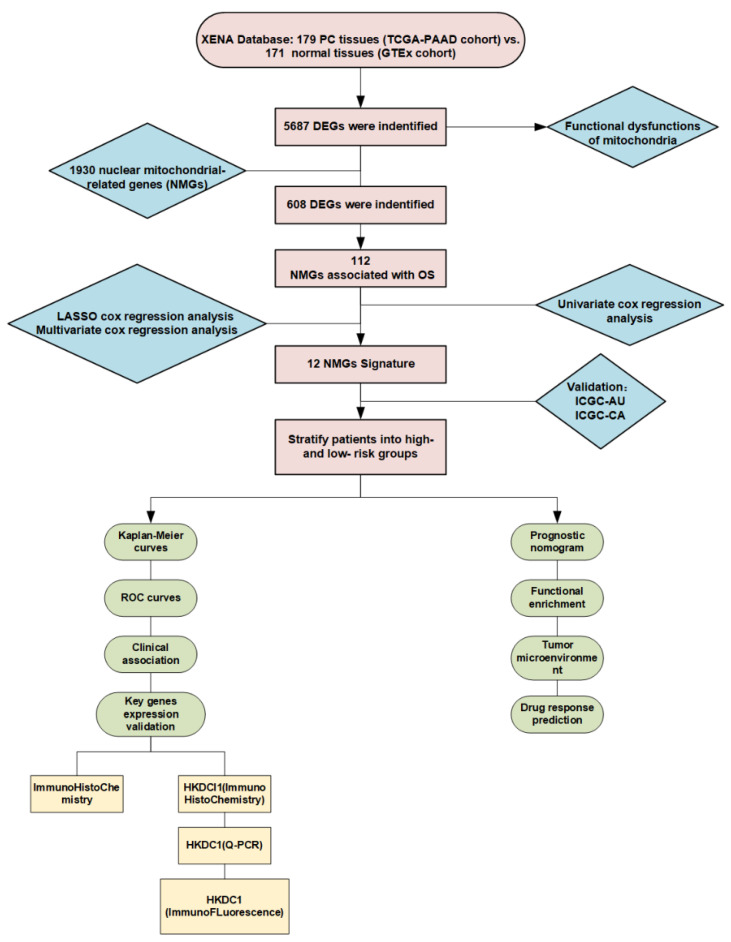
Flow chart of the overall research regarding nuclear mitochondria-related genes in PC.

**Figure 2:**
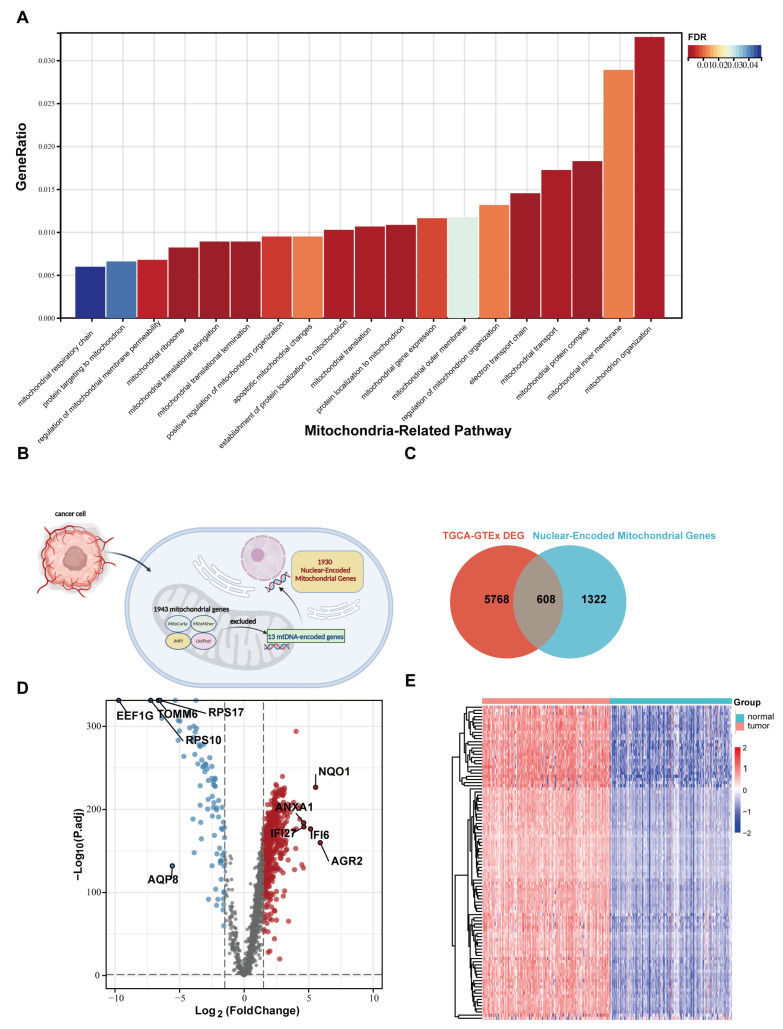
The analysis of mitochondrial dysfunction and nuclear mitochondria-related genes in PC. (**A**) Important mitochondria-related biological processes in the enrichment pathway of pancreatic cancer compared with the normal tissue. (**B**) The list of nuclear mitochondria-related genes determined by four datasets. (**C**) The Venn diagram indicates the interaction of nuclear mitochondria-related differential genes (nmDEGs) and pancreatic-cancer-related DEGs (PC−Normal DEGs). (**D**) The volcano plot displays the quantity of nuclear mitochondria-related DEGs between PC tissue and normal pancreatic tissue. (**E**) The heapmap shows the distribution of expression of nuclear mitochondria-related differential genes (nmDEGs).

The workflow of this study is shown in Figure 1. First, we preliminarily screened out the DEGs between PC tumor tissues and normal pancreatic tissues with functional enrichment analysis. We found that multiple biological pathways related to mitochondria played essential roles in PC development, as shown in Figure 2A. The clinicopathological features of 179 PC patients from the TCGA cohort are shown in Table 1.

### 2.2. Nuclear Mitochondria-Related Genes (NMGs) Are Significantly Changed in PC

By integrating information from four databases (the MitoCarta, MitoMiner, IMPI2, and UniProt databases), we obtained a list of 1943 mitochondrial genes. After removing 13 mtDNA-encoded genes, we finally obtained a list of 1930 nuclear mitochondria-related genes (NMGs) (Figure 2B). Using ANOVA, 608 nmDEGs were identified between PC tumor tissue and normal pancreatic tissue (Figure 2C). To further understand the effect of differentially expressed nmDEGs on biological activity, GO and KEGG enrichment analyses were performed (Supplementary Figure S1). In enrichment analysis for upregulated nmDEGs, multiple metabolism-related pathways were enriched, including fatty acid synthesis and degradation, carbon metabolism, the tricarboxylic acid cycle, and glycolysis. Energy-related pathways such as oxidative phosphorylation, electron transport chain, and ATP metabolic process also showed positive enrichment. The ferroptosis pathway was also positively enriched. Moreover, pathways related to mitochondrial structure and morphological remodeling were significantly enriched. Considering that the number of upregulated nmDEGs was much larger than that of downregulated NMGs, upregulated nmDEGs were more functionally diverse than downregulated nmDEGs.

### 2.3. An Innovative Prognostic NMG-Signature for PC

**Figure 3:**
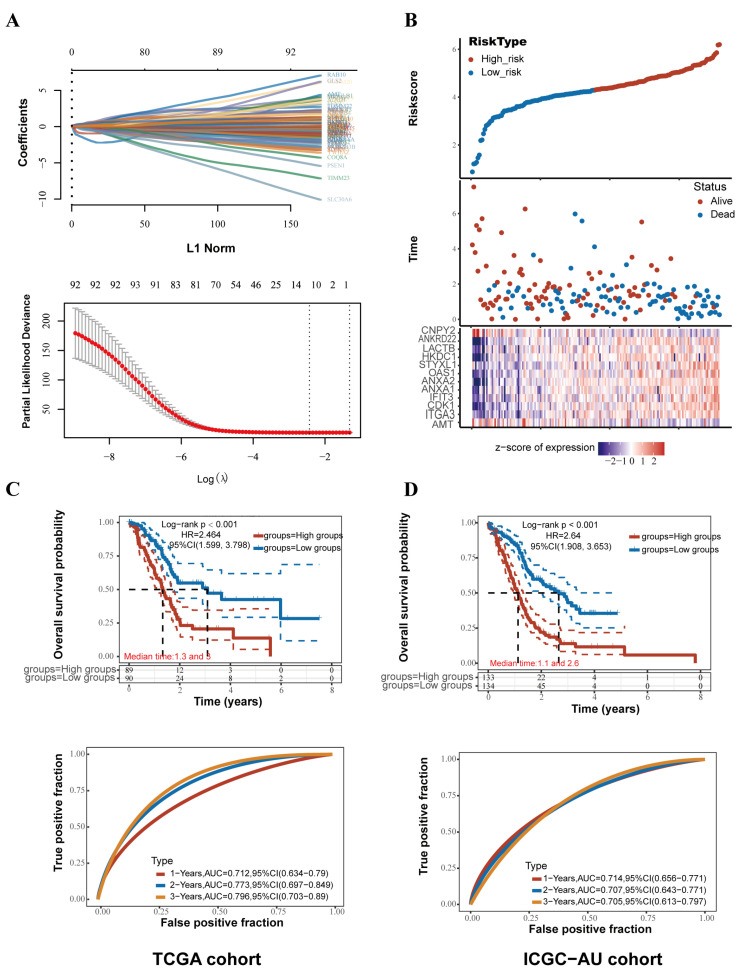
Construction and validation of the nuclear mitochondria-related gene signature. (**A**) LASSO Cox regression analysis determined a total of 12 NMGs as the optimal combination for the NMG signature construction. (**B**) Distribution of the risk score, overall survival, and survival status of the prognostic signature in the TCGA–PAAD cohort. (**C**) Kaplan–Meier survival curve for overall survival grouped by the median risk score and time-dependent ROC curves measuring the predictive value of the risk score in the TCGA–PAAD cohort. (**D**) Kaplan–Meier survival curve for overall survival grouped by the median risk score and time-dependent ROC curves measure.

We screened 112 prognosis-related nmDEGs using univariate Cox regression analysis. Through further LASSO regression analysis, 12 NMGs were identified, AMT, ITGA3, CDK1, ANKRD22, IFIT3, ANXA1, ANXA2, OAS1, STYXL1, HKDC1, CNPY2, and LACTB (Figure 3A). Then, through multiple stepwise Cox regression analyses, the prognosis-related risk score for PC patients was calculated with the following formula:

Risk score = (−3.2163) × AMT + (0.1613) × ITGA3 + (0.2622) × CDK1 + (0.3034) × IFIT3 + (−0.1363) × CNPY2 + (0.0357) × ANXA1 + (0.3008) × ANKRD22 + (0.1511) × ANXA2 + (−0.1393) × OAS1 + (0.5654) × STYXL1 + (0.0731) × HKDC1 + (0.0311) × LACTB

### 2.4. Survival Analysis and Validation of the NMG Signature

According to the median risk score, 179 PC patients were divided into a high-risk group and a low- risk group. Kaplan-Meier (K-M) survival analysis showed that the high-risk group had significantly worse OS (median OS: 1.3 vs. 3.0 years, *p* < 0.0001) than the low-risk group. Time-dependent ROC analysis was used to evaluate the predictive evaluation ability of the NMG signature. The AUC values at 1, 2, and 3 years for predicting OS were 0.712, 0.773, and 0.796, respectively (Figure 3C). Furthermore, two independent cohorts were retrieved to validate the NMG signature from the ICGC-AU (Figure 3D) and ICGC-CA cohorts (Supplementary Figure S2). Patients in the high-risk group had significantly worse OS in both external cohorts (ICGC-AU: median OS: 1.1 vs. 2.6 years, *p* < 0.0001; ICGC-CA: median OS: 1.0 vs. 1.8 years, *p* < 0.0001). The AUC values for predicting OS at 1, 2, and 3 years were 0.771, 0.771, and 0.797, respectively, in the ICGC-AU cohort (Figure 3D). The AUC values for predicting OS at 1, 2, and 3 years were 0.665, 0.723, and 0.714, respectively, in the ICGC-CA cohort (Supplementary Figure S2).

### 2.5. Comparison of Clinicopathological Features between the High- and Low-Risk Groups

**Figure 4:**
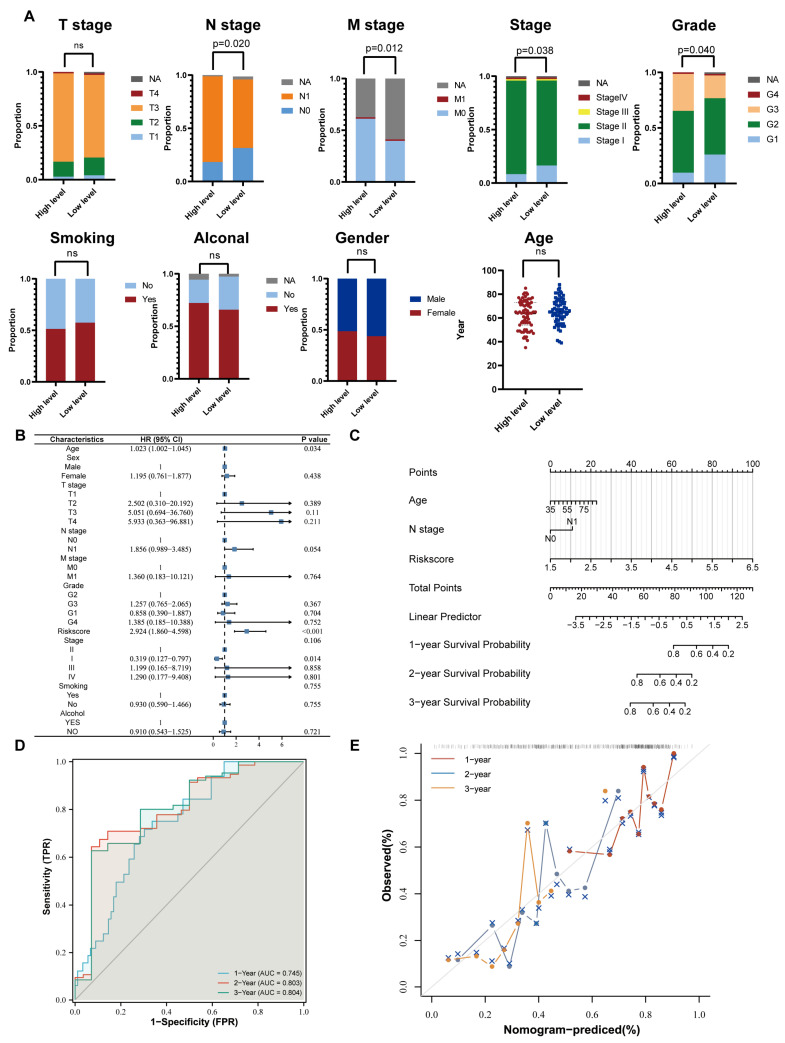
Comparison of clinical features between high- and low-risk groups and development of an OS predictive nomogram. (**A**) The percentage-staked bar plots and boxplot show the distribution of various clinical features between the high- and low-risk groups (“ns” stands for no significant difference between the values from the two groups.). (**B**) Multivariate Cox regression analysis for selection of primary clinical features and risk score correlated with overall survival of PC patients. (**C**) The NMG−based nomogram was constructed to predict the OS of PC patients. (**D**) The ROC curves of the nomogram for OS at 1, 2, and 3 years in the analysis of TCGA−PAAD cohort. (**E**) The calibration plots for the evaluation of predicted OS at 1, 2, and 3 years.

To further assess the diagnostic value of the NMG signature, we compared the different clinical characteristics of the high- and low-risk groups (Figure 4A). There was no statistically significant difference in terms of age, gender, alcohol, smoking, and T-stage. Significantly, we noticed that the high-risk group had a higher proportion of patients who developed lymph node metastasis, higher histological grading, and a more advanced TNM stage than the low-risk group. Notably, the differences between the two groups in M stage were unreliable and unanalyzable because there was too much information missing and a lack of M1-stage patients.

Overall, our risk model mainly distinguished the aggression-related characteristics of tumor cells rather than the attributes of patients to predict the prognosis of PC patients. Multivariate Cox regression model analysis was applied to further analyze the predictive value of these clinicopathological features (Figure 4B). Age, N-stage, and NMG risk scores were determined to be independent prognostic factors. A nomogram based on the selected prognostic factors was proven to perform well with AUC values of 0.745, 0.803, and 0.804 at the 1-, 2-, and 3-year timepoints, respectively (Figure 4C,D). The corresponding calibration curve also showed that the predicted survival value and actual survival value fitted well (Figure 4E). The above analysis proved the clinical application value of NMG signatures, which might be helpful for clinical decision making.

### 2.6. Identification of Differential Biological Functions

**Figure 5:**
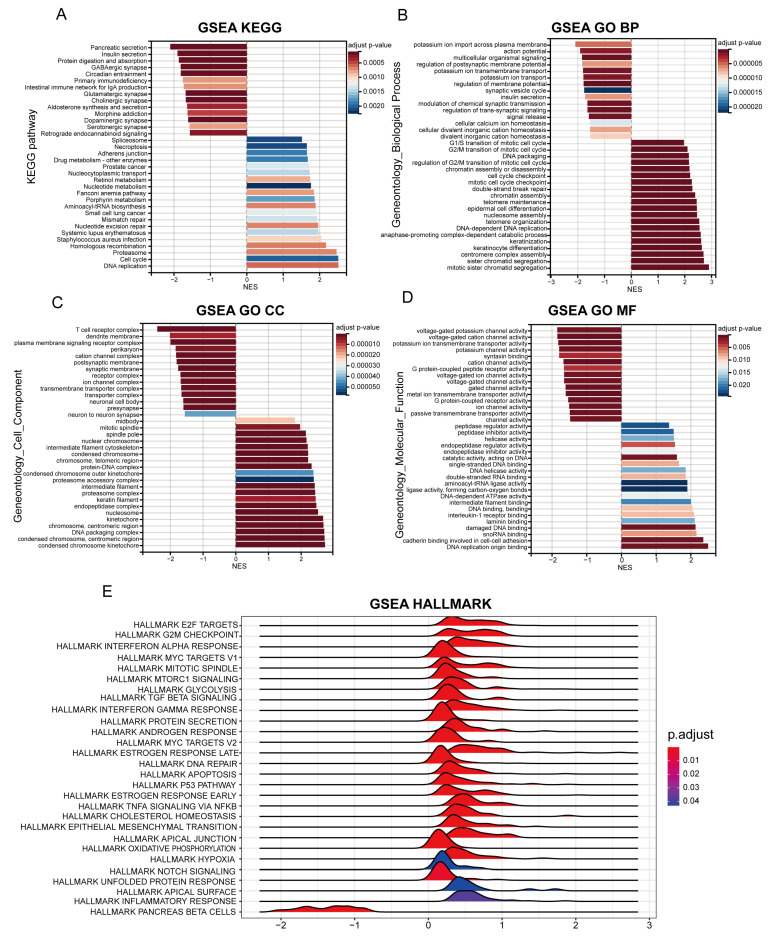
Gene set enrichment analysis (GSEA) between the high- and low-risk groups. (**A**) The KEGG pathway enrichment analysis. (**B**–**D**) The GO enrichment analysis including the biological process (BP), cellular component (CC), and molecular function (MF). (**E**) The HALLMARK gene set enrichment analysis. Note: *p* < 0.05 was considered statistically significant.

After differential expression analysis, 1274 genes were determined to be differentially expressed between the high- and low-risk groups, including 890 upregulated genes and 384 downregulated genes. To further understand the biological mechanisms associated with the two groups, GSEA based on GO, KEGG, and HALLMARK gene sets was conducted (Figure 5). The HALLMARK gene set enrichment analysis showed significant enrichment of multiple hallmark gene sets, including E2F targets, G2M checkpoint, interferon alpha response, and Myc targets (Figure 5E). The KEGG gene set enrichment analysis exhibited a significant abundance of spliceosome, necroptosis, adherent junction, drug metabolism, mismatch repair, cell cycle, and DNA replication (Figure 5A). The GO gene set enrichment analysis exhibited a significant abundance of spliceosome, necroptosis, adherent junction, drug metabolism, mismatch repair, cell cycle, and DNA replication (Figure 5B–D).

### 2.7. Genomic Features Associated with the NMG Signature

**Figure 6:**
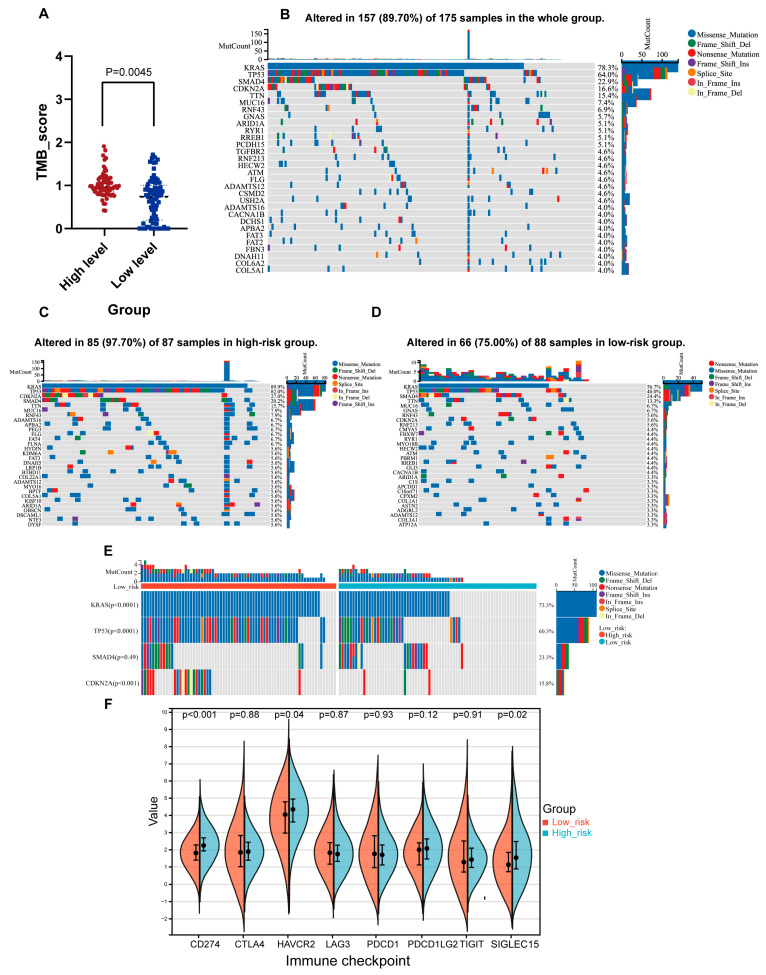
The analysis of genomic alterations between the high- and low-risk groups. (**A**) The boxplots show the tumor mutation burden between the high- and low-risk groups. (**B**) The overall genomic profiling of the top 30 most frequently altered genes in TCGA-PAAD cohort. (**C**) The genomic profiling of the top 30 most frequently altered genes in high-risk group cohort. (**D**) The genomic profiling of the top 30 most frequently altered genes in low-risk group cohort. (**E**) Comparison of the genomic profiling of four major genetic mutations of PC between the high-risk group and low-risk group. (**F**) Comparison of immune checkpoint expression levels between the high-risk group and low-risk group.

Then, we calculated the TMB levels of the two groups and found that the high-risk group showed a higher mutation burden than the low-risk group (Figure 6A). Comprehensive gene mutation profiles revealed that the frequency and type of altered genes between the high- and low-risk groups were distinct (Figure 6B–E). Overall, the high-risk group exhibited a higher mutation frequency in most genes, represented by KRAS (frequency: 89.9% vs. 56.7%, *p* = 0.05), TP53 (frequency: 82.0% vs. 40.0%, *p* < 0.001), SMAD4 (frequency: 27.0% vs. 24.4%, *p* = 0.05), and CDKN2A (frequency: 27.0% vs. 5.6%, *p* < 0.001). Considering the relationship between the tumor mutation landscape and immune checkpoint expression, we compared the immune checkpoint expression levels of the two risk groups (Figure 6F). We found that CD274 (*p* = 7.4 × 10^−5^), HAVCR2 (*p* = 0.04), and SIGLEC15 (*p* = 0.02) levels were increased in the high-risk group compared with the low-risk group.

### 2.8. Analysis of the Tumor Immune Microenvironment Associated with the NMG Signature

**Figure 7:**
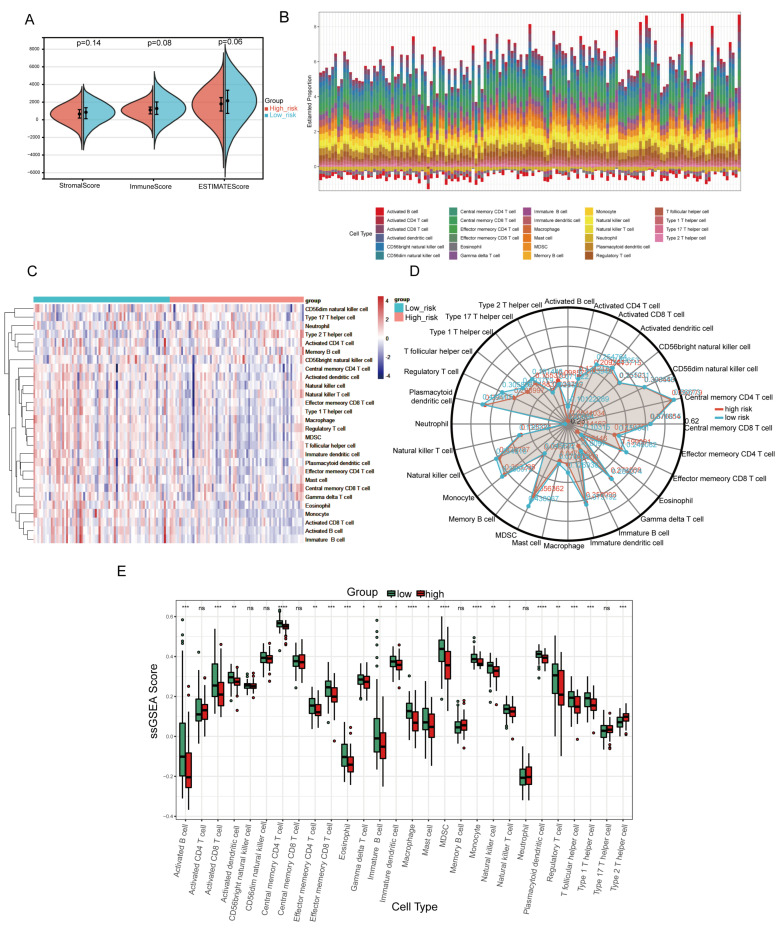
The analysis of tumor immune microenvironment between high-risk group and low-risk group. (**A**) The comparison of stromal score, immune score, and ESTIMATE score between the high- and low-risk groups. (**B**) A column stacking diagram exhibiting an overview proportion of different type of immune cell in TCGA cohort. (**C**,**D**) The heatmap and radar chart showing the difference in distribution of various immune cells between high- and low-risk groups. (**E**) The analysis of 28 immune infiltrated cells between the high- and low-risk groups. **** *p* < 0.0001, *** *p* < 0.001, ** *p* < 0.01, * *p* < 0.05. “ns” stands for no significant difference between the values from the two groups.

The stromal score, immune score, and ESTIMATE score were not significantly different between the two groups (Figure 7A). To evaluate the immune infiltration pattern of pancreatic cancer, ssGSEA was applied to assess the levels of different immune cell types according to the expression levels of immune-cell-specific marker genes (Figure 7B–E). The levels of most immune cell types declined in the high-risk group compared with the low-risk group, including activated CD8+ T cells, natural killer cells, and macrophages, whereas type II T helper cells exhibited a relatively high infiltration level in the high-risk group. Cell types including activated CD4+ T cells, CD56bight and CD56bright natural killer (NK) cells, central memory CD8+ T cells, memory B cells, neutrophils, and type 17 T helper cells showed no significant difference between the two risk groups.

### 2.9. Treatment Response Prediction of PC Chemotherapy

**Figure 8:**
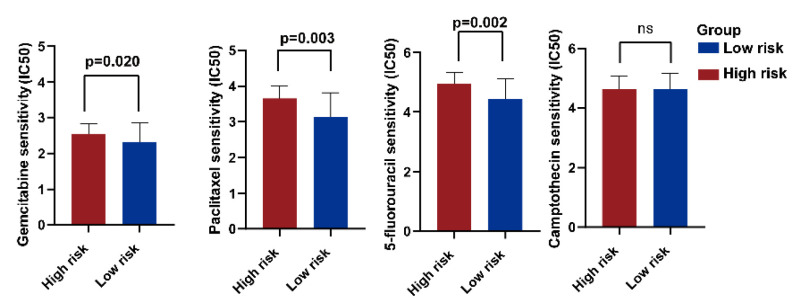
The predictive evaluation of treatment responses for several common chemotherapeutic drugs in PC by the novel prognosis score based on the NMG signature. “ns” stands for no significant difference between the values from the two groups.

The low-risk group showed more sensitivity to several common PC chemotherapy drugs, including gemcitabine, 5-fluorouracil, and paclitaxel, than the high-risk group. No noticeable difference was observed in the camptothecin response between the two groups (Figure 8).

### 2.10. Comprehensive Analysis of Key Genes in the NMG Signature

We analyzed the 12 key NMGs in the HPA database. All of them exhibited differential expression between PC tissue and normal pancreatic tissue. The protein expression of the AMT gene decreased in PC tissue compared with normal tissue, whereas the rest of the genes (AMT, ITGA3, CDK1, IFIT3, ANXA1, ANXA2, CNPY2, OAS1, STYXL1, HKDC1, LACTB, and ANKRD22) showed elevated protein levels in PC tissue compared with normal tissue (Supplementary Figure S3A,B). Next, we searched PUBMED for studies on the roles of the 12 key genes in pancreatic cancer. Some of them (ITGA3 [24], CDK1 [25], IFIT3 [26], ANXA1 [27], ANXA2 [28], OAS1 [29], and LACTB [30]) have been studied to varying degrees concerning their relationship with pancreatic cancer. Considering the background and potential functions of the remaining genes, we selected HKDC1 for further validation.

### 2.11. Validation of HKDC1 in the FUSCC Cohort

**Figure 9:**
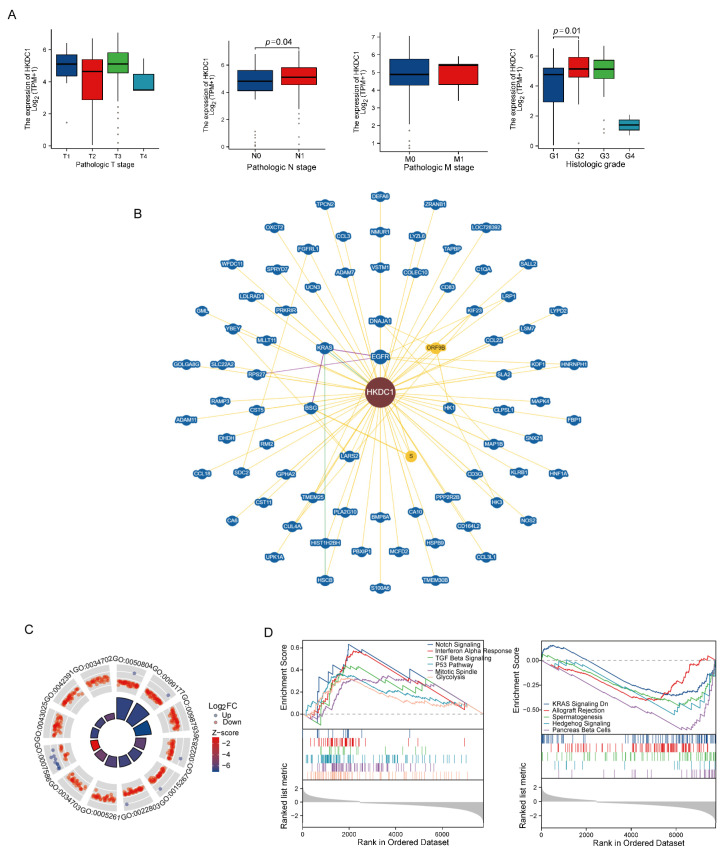
The predictive evaluation of treatment responses for several common chemotherapeutic drugs in PC by the novel prognosis score based on NMG signature. (**A**) The percentage-staked bar plots or boxplot show the distribution of primary clinical features between the high- and low-level HKDC1 groups. (**B**) The molecules interacting with HKDC1 obtained from BioGRID website. (**C**) The GO enrichment analysis of HKDC1 in PC. (**D**) Gene set enrichment analysis (GSEA) analysis of HKDC1 in PC.

**Figure 10:**
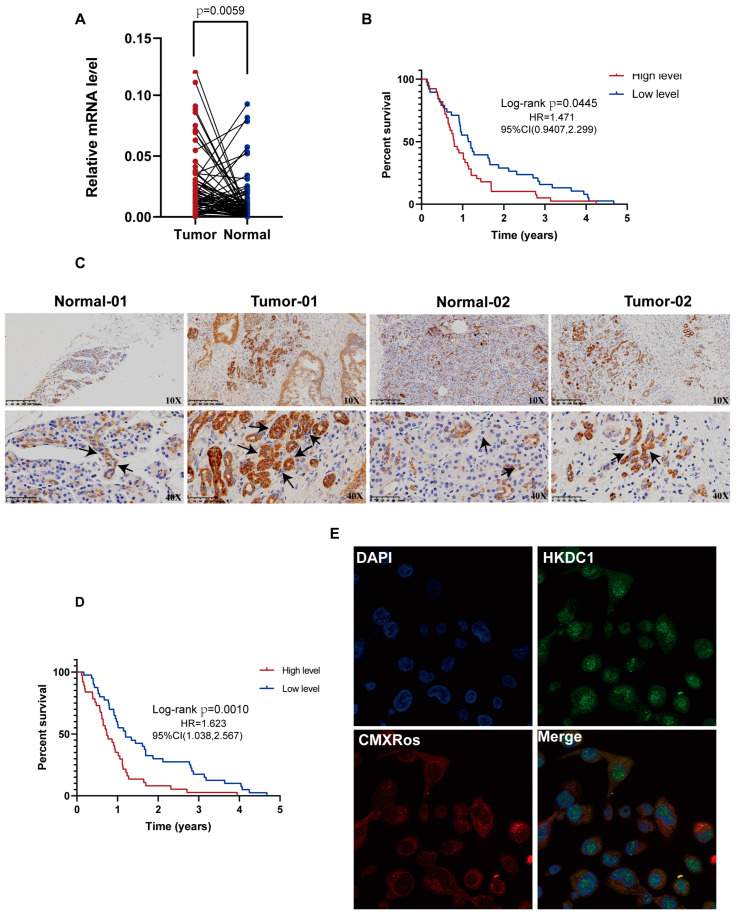
External expression validation of the selected mitochondrial gene, HKDC1. (**A**) The comparison of HKDC1 mRNA levels measured by qRT-PCR between 77 paired PC tumor tissues and normal tissues adjacent to cancer from our FUSCC cohort. (**B**) The Kaplan–Meier analysis of OS in different groups classified by the median value of HKDC1 mRNA levels in FUSCC cohort. (**C**) Representative microphotographs of HKDC1 staining in tumor (arrow) and matched adjacent normal tissues (arrowhead). (**D**) Survival analyses for OS of patients from FUSCC cohort with different HKDC1 expression levels in the tumor. (**E**) Immunofluorescence colocalization indicated that HKDC1 localized to mitochondria.

For the selected gene, HKDC1, we analyzed the different clinical characteristics between two groups divided by HKDC1 RNA expression level (high-level and low-level expression). The high-risk group had a higher proportion of patients who developed lymph node metastasis and higher histological grading (Figure 9A). Then we performed the functional enrichment analysis of HKDC1-related genes in pancreatic cancer. As shown in Figure 9B, the interacting molecules with HKDC1 were obtained from the BioGRID web tool. GSEA analysis revealed the possible signaling pathways related to HKDC1 (Figure 9C,D). We performed more validations in our cohort, the FUSCC cohort. This cohort included 77 samples from patients who were diagnosed with pancreatic cancer and underwent radical surgery from 2014–2016. The mRNA level of HKDC1 was significantly evaluated in PC tissue compared with normal tissue via Q-PCR (Figure 10A). The patients were divided into a high-level group and a low-level group according to the median value of mRNA levels. The Kaplan-Meier (K-M) plot validated the survival difference between the two groups (Figure 10B). Then, the protein level of HKDC1 was examined through immunohistochemistry. Although normal tissues also expressed HKDC1 protein, HKDC1 protein expression was more evident in PC tissues, mainly located in tumor cells rather than in the stroma (Figure 10C). According to the IHC scores, the samples were dichotomized into two groups (high-level and low-level) according to the median value. The high-level group exhibited poorer survival outcomes than the low-level group (Figure 10D). To determine the intracellular localization of HKDC1, we performed immunofluorescence to mark the HKDC1 protein with green fluorescence. Then, one type of mitochondrial probe, MitoTracker Red CMXRos, was used to label mitochondria red; the colocalization of HKDC1 with mitochondria is shown in the results (Figure 10E).

## 3. Discussion

Pancreatic cancer is a highly aggressive disease with a poor survival outcome. Difficulties in the early diagnosis and treatment strategy decisions of PC indicate a need for more effective biomarkers. Mitochondria, an essential cytoplasmic organelle, are responsible for energy production metabolism regulation, cellular apoptosis, and calcium homeostasis. Increasing evidence indicates that mitochondrial dysfunction can mediate PC progression through multiple mechanisms. PC cancer cell changes regulate the quantity and quality of mitochondria to better adapt to hypoxic, low pH, and nutrient-deficient microenvironments: on the one hand, KRAS induces mitophagy and reduces the total number of mitochondria via accumulation of one kind of protein called NIX, with the aims of eliminating excessive intracellular reactive oxygen species (ROS) and providing nutrient metabolites (e.g., glucose and glutamine) to support cell proliferation [31]. On the other hand, PC cells promote abnormal division and aberration of the mitochondrion to generate energy fuel more efficiently [32]. Mitochondria-mediated redox imbalance and calcium influx also play vital roles in the chemoresistance of pancreatic cancer [23]. Through functional enrichment analysis between PC and normal tissues, we found that multiple mitochondria-associated signaling pathways and biological processes were considerably changed, indicating the vital effect of mitochondria in PC development.

Mitochondria contain their own genome in the form of mitochondrial DNA (mtDNA), which encodes 13 essential proteins of mitochondrial respiratory chains and OXPHOS complexes, 2 ribosomal RNAs (mt-rRNAs), and 22 transfer RNAs (mt-tRNAs) [33]. Compared with the normal pancreas, the mtDNA copy number in PC cancer tissues is significantly reduced, and mtDNA content is negatively correlated with the malignancy of PC [34]. Accumulation of mtDNA variations may facilitate the susceptibility and metastasis of pancreatic cancer [35]. However, their role in the progression of pancreatic cancer is still unclear and more research needs to be performed.

Significantly, most mitochondrial proteins are encoded by the nuclear genome [36]. After synthesis on cytoplasmic ribosomes, the precursors of NMGs are localized to mitochondria with the guidance of a leader peptide. They are messengers between the nucleus and mitochondria, determining more mitochondrial functions than mtDNA-encoding proteins. Given the diversity of the types and functions of NMGs, we conducted a comprehensive analysis to investigate the transcriptional landscape differences between PC tissue and normal tissues.

Through bioinformatic analyses, we found that a large proportion of NMGs were changed in PC compared with normal tissue (1120/1930). These altered NMGs were sprinkled throughout all kinds of material and energy metabolic processes (generation and degradation of ATP, fatty acid metabolism, glycolysis, oxidative stress response, and so on) and cell death modes (autophagy and ferroptosis), influencing PC development with a remarkable scope and degree. 

Oxidative stress is the redox state resulting from an imbalance between ROS accumulation and detoxification. As the major producer of intracellular ROS, the mitochondrion is considered to be the primary target for oxidative stress in cancer [37]. The role of oxidative stress in cancer development is double-edged and varies depending on the different dosages, time periods, and cellular distributions [38]. ROS could directly result in DNA damage and mutations of different types that drive the occurrence of tumors. In particular, the oxidative stress-induced responses happening in mitochondria also alter the mutational and epigenetic landscape of mtDNA, causing mitochondrion dysfunction and generating more ROS [39]. This regulation also influences mutual regulation between nuclear DNA (nDNA) and mtDNA. Chronic moderate to low level does of ROS could promote cell mitosis and proliferation, with increasing genomic instability of the newly generated cells, whereas acute and high-concentration ROS may alter the structure of biological macromolecules including proteins, lipids, and nucleic acids, causing cell necrosis and apoptosis [40,41,42]. Metabolism reprogramming, as a vital hallmark of the cancer, often increases the intracellular levels of ROS in the tumor, whereas tumor stem cells and drug-resistant cells exhibit a relatively low level of ROS because of the protective antioxidant protein expression. Considering the complicated functions of ROS in tumor progression, the therapeutic strategies targeted at oxidative stress are also controversial. Previous researchers have not reached a unified conclusion about the value of the antioxidants in clinical application: the antioxidants could exhibit pro- or anti-cancer effects in aspects of the cancer prevention and adjuvant treatment effect influenced by various dosages, nutrient types, and tumor heterogeneity [43,44,45,46,47]. One possible explanation for the pro-cancer effect of the antioxidants is that the drugs unable to enter the intracellular mitochondria, accumulate outside and change the oxidative balance of the normal cells [48]. Therefore, targeting mitochondrial oxidative stress, including the mitochondria-located proteins, seems a promising treatment method. Wang’s team development a small molecule agonist ZG111 of the mitochondrial caseinolytic protease P (ClpP) that produces tumor inhibitory effects in pancreatic cancer cell lines and mouse models [49]. This study further proved that differentially expressed mitochondria-related genes are active in the oxidative stress pathway. Based on the NMGs, the established 12-gene signature well stratified different groups of PC patients not only in the field of prognostic prediction but also in immune infiltration and drug resistance.

Immunotherapy, which activates the immunoreactive system in vivo for combatting malignancies, has revolutionized traditional tumor therapeutic methods [50]. Immunotherapy represented by immune checkpoint inhibitors has demonstrated clinical benefit in many solid tumors [51]. However, no significant survival improvement was observed for immunotherapies in pancreatic cancer, potentially because of the low tumor mutation burden in PC [52]. Tumor mutational burden (TMB) is defined as the number of somatic, coding, base substitution, and indel mutations per megabase of genome examined, which is a typical biomarker of ICIs [53]. We found that the high-risk group had a relatively high TMB level compared with the low-risk group, indicating a more promising effect for ICIs in the high-risk group. Then, we further compared the mutational landscape in the two groups, especially the four major genetic mutations of pancreatic cancer, namely, those in KRAS, p53, CDKN2A, and SMAD4/DPC4.2 [54]. Notably, the high-risk group exhibited a significantly higher mutational prevalence in KRAS, TP53 and CDKN2A and a lower mutational prevalence in SMAD4 than the low-risk group. As we mentioned before, the KRAS gene may be an important driver of mitochondrial remodeling in pancreatic cancer. TP53 is a gene that helps stop the growth of tumors. The TP53 gene encodes the p53 protein, which is a tumor suppressor that prevents cell division and proliferation. P53 induces transcription-independent apoptosis through a mitochondrial pathway [55]. SMAD4 is a crucial tumor suppressor gene and is frequently subverted in cancer progression. Ezrova et al. proved that SMAD4 deficiency could promote the drug resistance of PC cells to mitochondria-targeted metformin (MitoMet) via TGFβ signaling and mitophagy [56]. Considering the relationship between the tumor mutation profile and immune checkpoint inhibitors, we examined the mRNA levels of several typical immune checkpoints (CD274, CTLA4, HAVCR2, LAG3, PDCD1, PDCD1LG2, TIGHT, and SIGLEC15) and found that the high-risk group had increased expression of some specific immune checkpoints, including CD274, HAVCR2, and SIGLEC15.

Next, we further investigated the discrepancy in the tumor microenvironment and immune infiltration between the two groups. The high-risk group exhibited lower infiltration levels in the bulk of the immune cell types (except for type II T helper cells) than the low-risk group. Previous researchers have found that type II helper cells infiltrate the pancreas early during tumorigenesis and drive PC progression by facilitating glycolysis and generating IL-4 and IL-13 [57]. Monte et al. verified the elevated TH2/TH1 ratio in pancreatic cancer tissue compared with normal pancreatic tissue by immunohistochemistry [58]. Additionally, the ratio of infiltrating TH2 to TH1 cells and the circulating levels of IL-4 in PC patients are negatively correlated with survival [59]. Interestingly, mitochondrial reactive oxygen species could induce the activation of primary human T cells by regulating IL-2 and IL-4 expression [60]. GATA3, a cell-specific marker of TH2 cells, could modulate mitochondrial biogenesis and increase the mitochondrial content [61,62]. The abovementioned studies show that mitochondrial regulation, cell metabolism, and oxidative stress may play certain roles in the differentiation and activation of TH2 cells, which may be related to the difference in TH2 cell infiltration between the high- and low-risk groups. However, the specific mechanisms need to be further explored in detail in the future. Combined with the immune checkpoint analysis above, we speculated that the NMG signature marked a relatively immunosuppressed cohort as the high-risk group that may be close to the concept of a “cold tumor”. For the high-risk group, immune checkpoint inhibitors targeting CD274, HAVCR2, and SIGLEC15 may produce a marked effect, whereas in the low-risk group, purposefully removing certain types of immunosuppressive cells, such as regulatory T cells and M2-type macrophages, would be more promising.

Because most patients have lost the chance of curative surgery at the time of diagnosis with PC, it is essential to develop nonsurgical therapeutic approaches. Chemotherapy is still the primary nonoperative therapeutic strategy for pancreatic cancer, but drug resistance is a severe challenge [63]. The low-risk group showed more sensitivity to several common PC chemotherapy drugs, including gemcitabine, 5-fluorouracil, and paclitaxel, than the high-risk group, whereas no obvious difference was observed in the response to camptothecin between the two groups. Notably, although the GDSC database is an invaluable tool in drug resistance prediction, most of the relevant information comes from cell lines and has not been demonstrated in cancer tissue. Therefore, further confirmation by subsequent specific experiments is required to verify the correlation between chemotherapy resistance and the NMG signature.

We further investigated the genes in the NMG signature (AMT, ITGA3, CDK1, IFIT3, ANXA1, ANXA2, CNPY2, OAS1, STYXL1, HKDC1, LACTB, and ANKRD22). Some genes have been explored in tumors or pancreatic cancer. The genes that are still relatively lacking in current research studies are AMT, CNPY2, STYXL1, HKDC1, LACTB, and ANKRD22. Among these unknown genes, the HKDC1 gene attracted our attention. Hexokinase domain containing 1 (HKDC1) was the fifth recently discovered hexokinase. The hexokinase family includes HK1, HK2, HK3, GCK (glucokinase, HK4), and HKDC1, which play important roles in glycolysis metabolism and cell fate. There are several reports regarding the function of HK1, HK2, and GCK in pancreatic cancer [64,65,66,67]. Therefore, we selected HKDC1 as the first gene for further validation. RT-qPCR proved that HKDCI was expressed at a higher level in PC tissue than in normal pancreatic tissue. Western blot analysis showed the high expression level of HKDC1 in several common pancreatic cancer cell lines. Immunohistochemistry revealed HKDC1 expression at the protein level and its positive correlation with poor prognosis. Immunofluorescence showed that HKDC1 was localized in the mitochondria. We are establishing related stable cell lines to conduct further exploration.

Nonetheless, there are still some limitations in our present research. Our study was primarily based on retrospective data, and further verification in laboratory experiments and prospective studies is needed. Conversely, genes from the mitochondrial genome were not included in the survey because relevant transcription data are lacking. We have been constantly collecting clinical samples to validate the NMG signature and constructing stable cell lines to further research the precise mechanisms of key genes. We hope our study will broaden the scope of the mitochondrial theory in pancreatic cancer, providing valuable ideas for future researchers and help with clinical decisions.

## 4. Materials and Methods

### 4.1. Datasets and Online Tools

The RNA sequencing expression profiles, genetic mutations, and matched clinical information for 179 PC patients were acquired from the Pancreatic Adenocarcinoma (TCGA-PAAD) cohort, and the gene expression data of 171 normal pancreatic tissue samples were collected from The Genotype-Tissue Expression database (GTEx). The ICGC-PACA (namely, ICGC-AU and ICGC-CA) cohorts with 260 patients were used as two external independent validation datasets, whose datasets were downloaded from International Cancer Genome Consortium (ICGC, https://dcc.icgc.org, accessed on 13 November 2022) The chemotherapy-related information was obtained from the most prominent public pharmacogenomics database, Genomics of Drug Sensitivity in Cancer(GDSC, https://www.cancerrxgene.org/, accessed on 11 November 2022). The protein expression and distribution of the key genes were exhibited via the IHC images acquired from the Human Protein Atlas (HPA, https://www.proteinatlas.org, accessed on 13 November 2022). The Human Protein Atlas (the HPA database) is an integrative multi-omics database containing large volumes of protein expression data in normal and cancerous tissues in the form of immunohistochemical imaging [68,69]. All the data can be freely accessed from the online website: https://www.proteinatlas.org. The Biological General Repository for Interactionh Datasets (BioGRID, https://thebiogrid.org, accessed on 13 November 2022) website was used to analyze the protein–protein interaction network [70].

The list of nuclear mitochondria-related genes (NMGs) was obtained from the MitoCarta (Version 3.0) [36], MitoMiner (Version 4.0) [71], IMPI2, and UniProt databases [72].

### 4.2. Screening Out the Differentially Expressed Genes in PC

Integrated transcriptome data from 179 PC tumor tissues (TCGA-PAAD cohort) and 171 normal pancreatic tissues (GTEx cohort) were exported online from the UCSC XENA database (UCSC, https://xena.ucsc.edu/, accessed on 12 November 2022) with the batch effect removed. Differentially expressed genes (DEGs) between PC and normal pancreatic tissue (PC-Normal DEGs) were determined via the “limma” package for R statistical software. The criteria for the identification of DEGs were an adjusted *p*-value < 0.05 and an absolute log2-fold change > 1.5. The intersecting genes in PC-Normal DEGs and NMGs were established as nuclear mitochondria-related differential genes (nmDEGs) and shown by Venn diagram. The visualization of nmDEGs was displayed in the form of a volcano plot and heatmap using the “ggplot” package.

### 4.3. Functional Enrichment Analysis

Kyoto Encyclopedia of Genes and Genomes (KEGG) analysis was performed to determine the primary biological actions of nmDEGs. Gene Ontology (GO) analysis, including three categories: biological process (BP), cellular component (CC), and molecular function (MF), was utilized to find potential targets of nmDEGs based on the “ClusterProfiler” package in R software (version 4.0.5; The R Foundation). Colors indicate the significance of functional enrichment, and the size of the circles represents the number of DEGs. The selective criterion for enrichment results was a *p* value less than 0.05.

### 4.4. Establishment of the Mitochondria-Related Gene Signature 

First, the OS-associated nmDEGs were identified via univariate Cox regression. Next, least absolute shrinkage and selection operator (LASSO) regression was used to minimize overfitting and identify the most significant survival-associated nmDEGs in PC via the “glmnet” package. Meanwhile, the corresponding coefficients were calculated by multivariate Cox regression analysis. In this process, the training set was the TCGA-PAAD cohort and ICGC-AU and ICGC-CA were used as two external validation cohorts. The formula combining the coefficient with the selected gene expression levels was used to determine the prognosis-related risk score. Based on the median value of the risk score, samples above this median were classified as the high-risk group, and those below this median were classified as the low-risk group. Kaplan–Meier and receiver operating characteristic curves were drawn to show the difference in survival probability of various risk groups.

### 4.5. Comparison of Pathological Features between Low- and High-Risk Groups and Establishment of the Prognostic Nomogram

We further analyzed the main clinical features, including age, sex, histological grade, clinical stage, TNM stage, alcohol consumption, and smoking, together with the prognosis score of the nmDEG signature through univariate and multivariate Cox regression analyses. After independent prognostic factors were determined, a survival-related nomogram was developed based on the selected variables. The time-dependent ROC curve and calibration curve were plotted to evaluate the predictive performance of the novel nomogram. The R packages included the “rms”, “survival”, and “timeROC” packages.

### 4.6. Gene Set Enrichment Analysis (GSEA)

To determine changes in biological effects and related pathways associated with risk score groups, gene set enrichment analysis (GSEA) was performed between the high- and low-risk groups. Compared with regular GO/KEGG enrichment analysis, GSEA is a quantitative method and includes more information about the expression of more synergistic genes. GSEA normalized the enrichment score for each gene set to account for the variation in gene set sizes, yielding a normalized enrichment score (NES). The gene sets we identified were as follows: GO KEGG (Kyoto Encyclopedia of Genes and Genomes) pathway, GO (Gene Ontology) enrichment analysis, and the HALLMARK gene set (including 50 gene sets from Molecular Signature Database, https://www.gsea-msigdb.org/gsea/msigdb/, accessed on 14 November 2022).

### 4.7. Tumor Gene Mutation Landscape Analysis

The mutation data were downloaded and visualized with the help of the R package “maftools”. Waterfall diagrams were drawn to exhibit the type and frequency of gene mutations in different risk groups. Immune-checkpoint-related gene expression differences in the two risk groups were compared via a grouped column chart.

### 4.8. Tumor Immune Microenvironment Analysis in PC

The stromal, immune, and ESTIMATE scores of the PC tumor tissues were determined using the ESTIMATE R package [73]. Immune cell type enrichment analysis was performed through single-sample gene set enrichment analysis (ssGSEA) [74]. The gene signatures of 28 tumor-infiltrating lymphocytes (TILs) were obtained from the TISIDB database (http://cis.hku.hk/TISIDB/download.php, accessed on 14 November 2022). Then, column stacking diagrams were plotted to exhibit an overview of the proportions of the different types of immune cells in every PC tissue. A heatmap, radar chart, and grouped column chart were drawn to show the differences in immune cell infiltration between the low- and high-risk groups.

### 4.9. The Evaluation of Precision Treatment and Chemotherapy Response

Prediction of the chemotherapeutic response for each sample was based on the largest publicly available pharmacogenomics database, the GDSC database. The R package “pRRophetic” was used to perform the prediction procedure. The half-maximal inhibitory concentration (IC50) of each case was calculated by ridge regression. Every parameter was set as the default value. The duplicate gene expression was summarized as a mean value considering the batch effect of combat and various tissue types.

### 4.10. Cell Transfection and Real-Time Quantitative Polymerase Chain Reaction (RT-qPCR)

Total RNA was extracted from 77 pairs of PC tissues from the Fudan University Shanghai Cancer Center (FUSCC) cohort with TRIzol reagent (Beyotime, Shanghai, China). RT-qPCR was then performed on a QuantStudio 6 system (Thermo Fisher Scientific, Waltham, MA, USA) using the Premix Pro Taq HS qPCR Kit (Accuate Biotechnology, Hunan, China) according to the manufacturer’s instructions. The fold-change value of mRNA was calculated by the 2−ΔΔCt method. The primers used were as follows: β-actin sense: TCATGAAGTGTGACGTGGACATC; β-actin antisense: CAGGAGGAGCAATGATCTTGATCT; HKDC1 sense: ACTGCACAGGAATCTCTGCC; HKDC1 antisense: CAGGAACCTGTCCACCTTCT.

### 4.11. Immunohistochemical (IHC) Staining and Analysis

IHC was performed with paraffin-embedded sections of PC tumor specimens. Primary antibodies composed of polyclonal rabbit antihuman HKDC1 (anti-HKDC1 [C- 4], Cat No. 25874-1-AP, diluted 1:200; Proteintech, Shanghai, China) were used. The staining scores for positive cells were estimated according to the following two aspects: the intensity scores (negative: 0, weak: 1, moderate: 2, and strong: 3) and the extent scores (0%: 0, 1–25%: 1, 26–50%: 2, 51–75%: 3,  >75%: 4). The positive scores were evaluated by two independent investigators blinded to the corresponding clinical information for each sample.

### 4.12. Immunofluorescence (IF) and Analysis

Immunofluorescence staining was performed on the pancreatic cancer cell line PANC-01 cultivated in confocal dishes. Primary antibodies composed of polyclonal rabbit antihuman HKDC1 (anti-HKDC1 [C-], Cat No. 25874-1-AP, diluted 1:100; Proteintech, Shanghai, China) were used. FITC-labeled Goat Anti-Rabbit IgG (H + L)) (Beyotime, Shanghai, China) was applied to further label the HKDC1 protein (green). DAPI was used to stain the nuclei (blue). IgG FITC-labeled anti-fluorescence MitoTracker Red CMXRos (Beyotime, Shanghai, China) was used to label bioactive mitochondria in cells (red). Observations were performed under a confocal microscope.

### 4.13. Statistical Analysis

R software (version 4.0.5; The R Foundation) was used for data calculations and picture drawing. GraphPad Prism 8.0 was used for statistical data analysis. Fisher’s test was implemented to compare differences in categorical variables. Kaplan-Meier curve analysis was executed to compare survival differences between two groups, and the log-rank test was used to assess the statistical significance of the survival rates among various risk groups.

## 5. Conclusions

Our research first systematically summarized the mitochondrial transcriptome characterization in pancreatic cancer and established a prognostic mitochondrial gene signature. The NMG signature showed good discrimination performance for survival rate and chemotherapy resistance prediction for PC patients. The high- and low-risk groups exhibited evident heterogeneity in the tumor mutation profile, immune infiltration, and chemotherapy sensitivity. Moreover, we certified the expression of some key genes at the mRNA and protein levels. Overall, this study will enrich the available therapeutic targets and research field for pancreatic cancer relevant to mitochondria.

## Figures and Tables

**Table 1:** Clinicopathological features of 179 patients from the TCGA−PAAD cohort.

Variables		Number	Percentage
Total		179	
Gender			
	Female	80	44.69%
	Male	99	55.31%
Age			
	≤65	94	52.51%
	>65	85	47.49%
Race			
	Asian	11	6.15%
	Black or African American	6	3.35%
	White	157	87.71%
	NA	5	2.79%
T stage			
	T1	7	3.91%
	T2	24	13.41%
	T3	142	79.33%
	T4	3	1.68%
	NA	3	1.68%
N stage			
	N0	50	27.93%
	N1	123	68.72%
	NA	6	3.35%
M stage			
	M0	79	44.13%
	M1	5	2.79%
	NA	95	53.07%
Pathologic stage			
	Stage I	21	11.73%
	Stage II	146	81.56%
	Stage III	3	1.68%
	Stage IV	5	2.79%
	NA	4	2.23%
Histologic grade			
	G1	31	17.32%
	G2	95	53.07%
	G3	48	26.82%
	G4	2	1.12%
	NA	3	1.68%
Smoker			
	No	65	36.31%
	Yes	79	44.13%
	NA	35	19.55%
Alcohol history			
	No	65	36.31%
	Yes	101	56.42%
	NA	13	7.26%

## Data Availability

Not applicable.

## Supplementary Materials

The following supporting information can be downloaded at: https://www.mdpi.com/article/10.3390/ijms24043270/s1.
