# Supplementary figures and images for: Mitochondria-Related Transcriptome Characterization Associated with the Immune Microenvironment, Therapeutic Response and Survival Prediction in Pancreatic Cancer
Source: Int J Mol Sci. 2023 Feb 7;24(4):3270. doi: 10.3390/ijms24043270 (PMC9966003; doi:10.3390/ijms24043270)

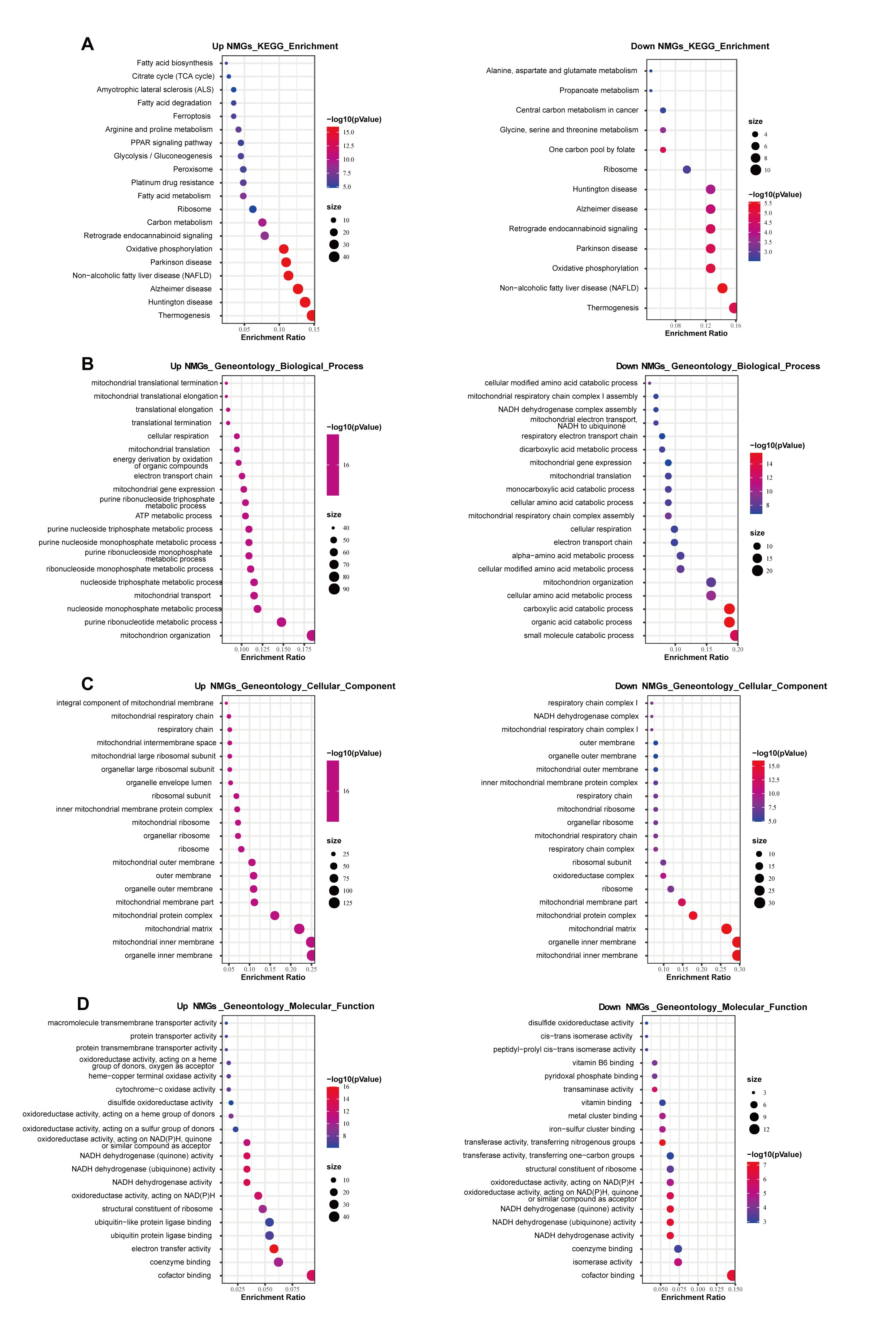

Supplement: Supplementary file 1 [file ijms-24-03270-s001.zip › supplementary figures/Supplementary Figure S1.tif]

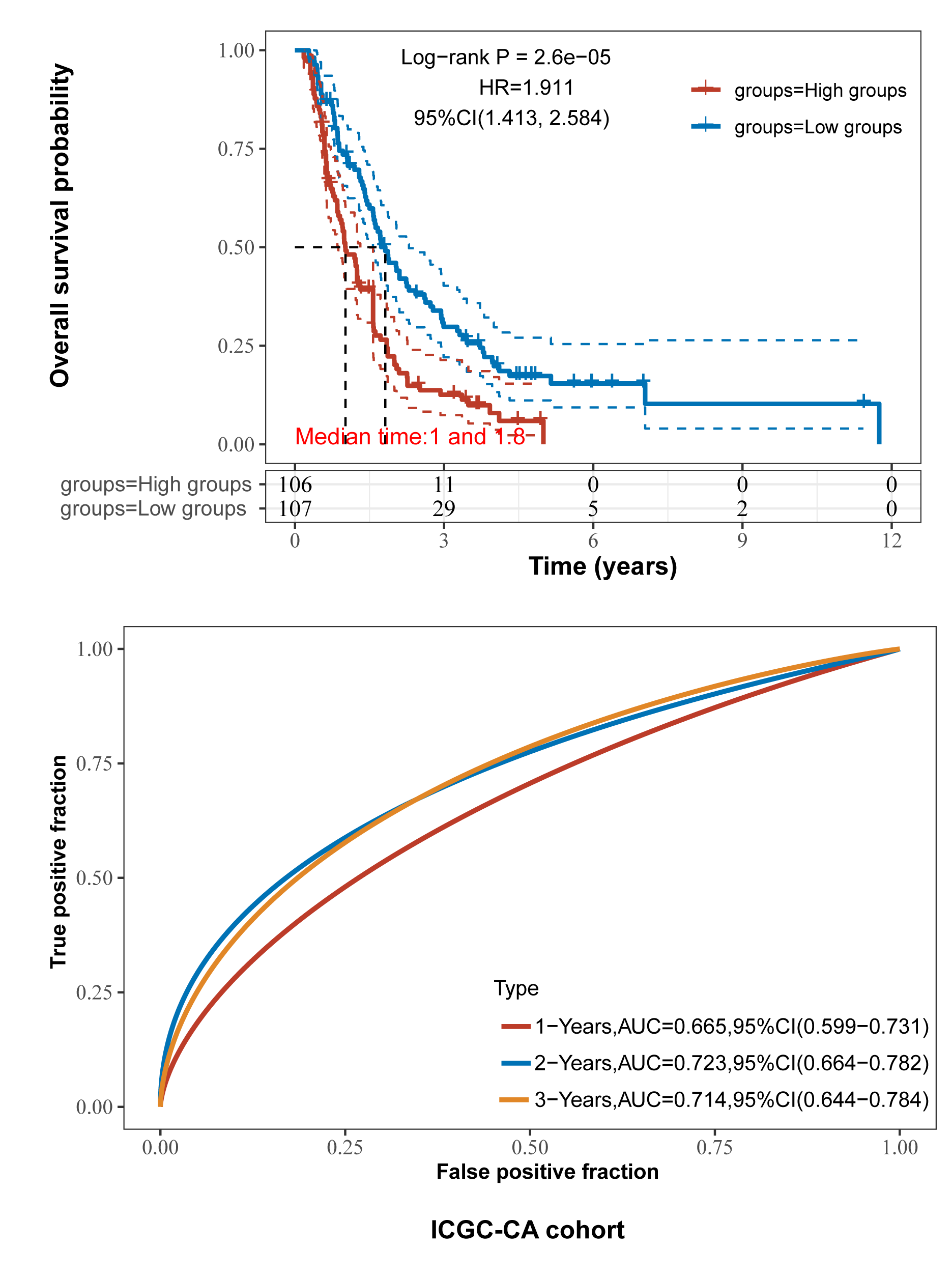

Supplement: Supplementary file 1 [file ijms-24-03270-s001.zip › supplementary figures/Supplementary Figure S2.tif]

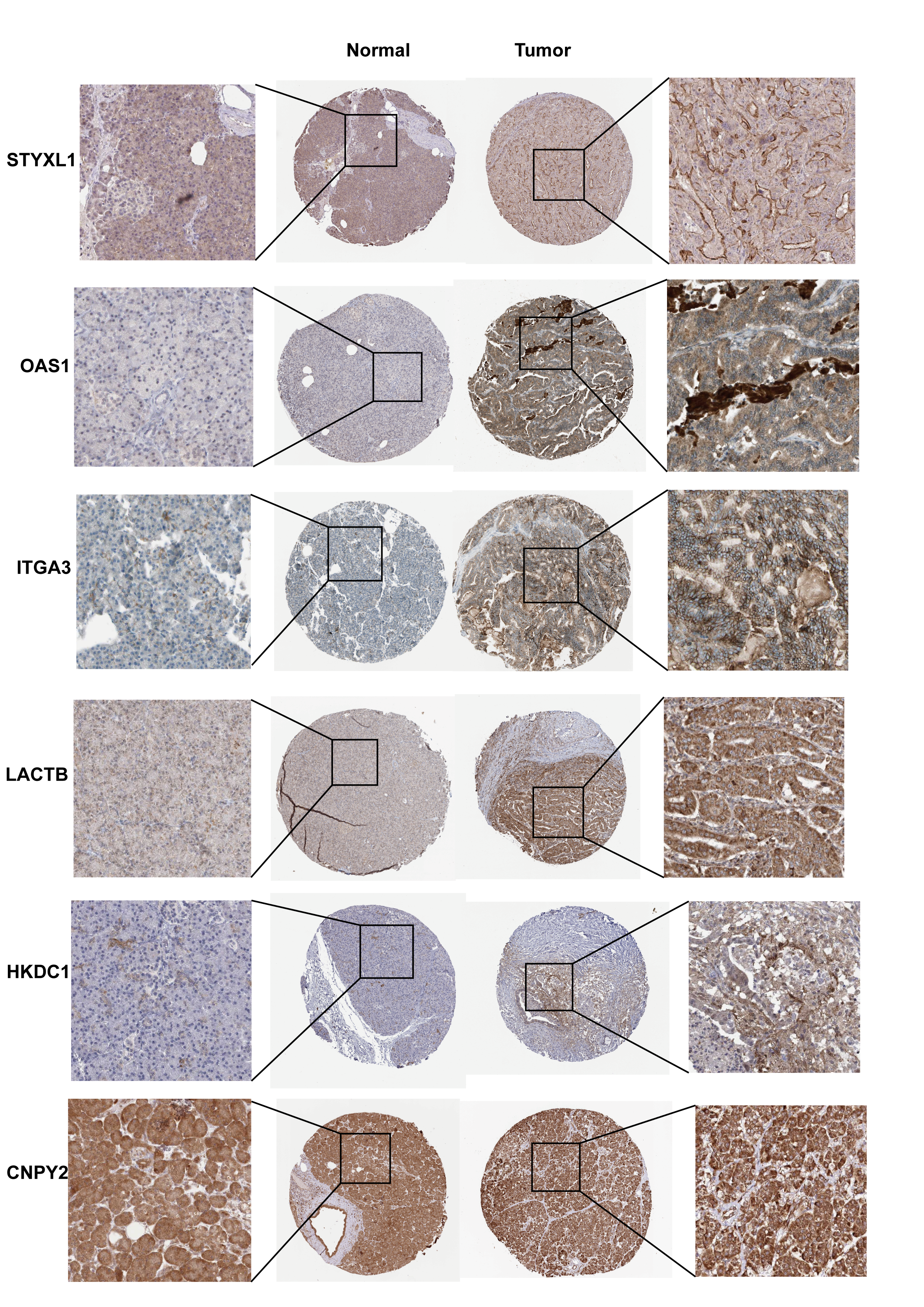

Supplement: Supplementary file 1 [file ijms-24-03270-s001.zip › supplementary figures/Supplementary Figure S3A.tif]

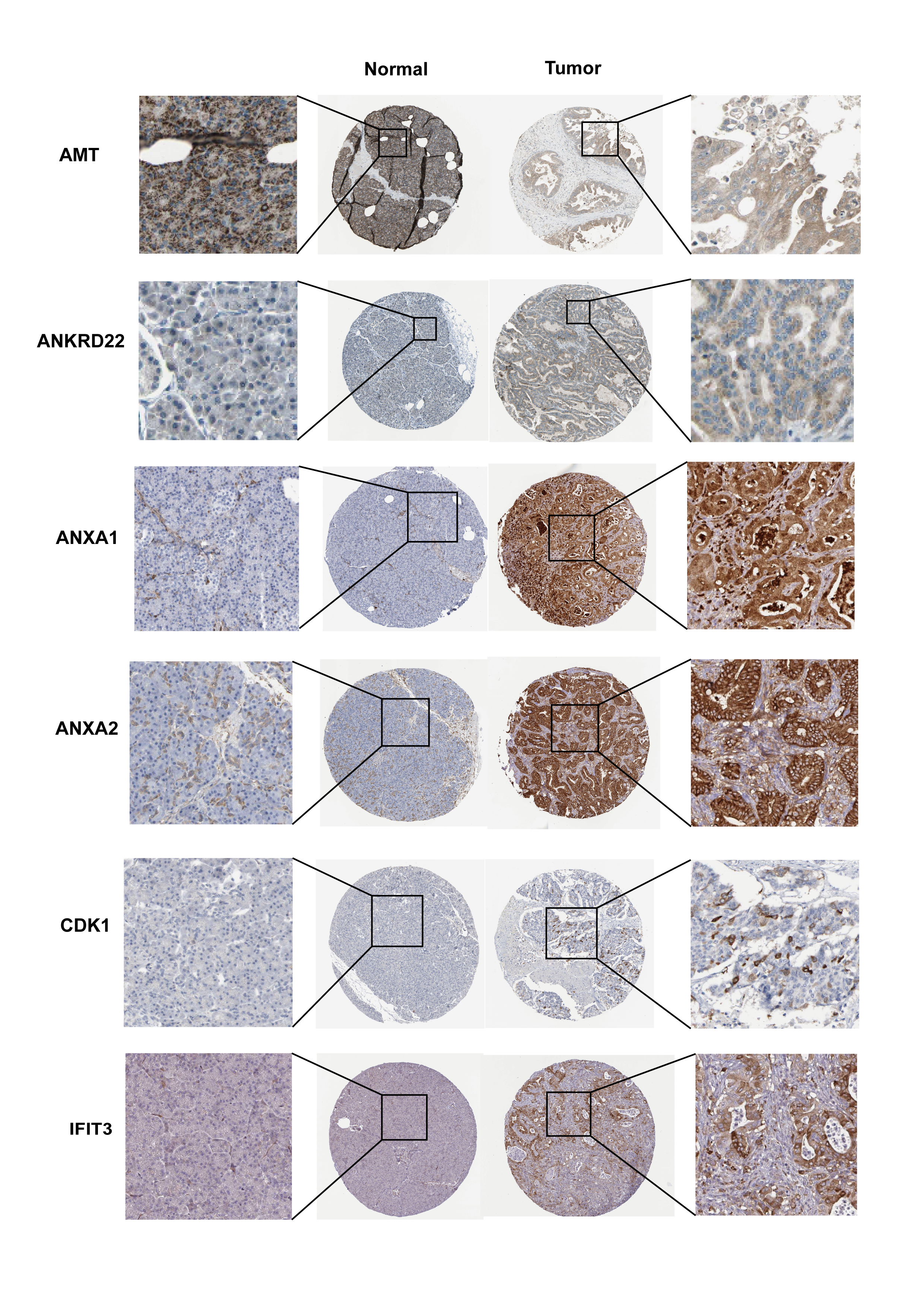

Supplement: Supplementary file 1 [file ijms-24-03270-s001.zip › supplementary figures/Supplementary Figure S3B.tif]
